# An Urologic Face of Chronic Lymphocytic Leukemia: Sequential Prostatic and Penis Localization

**DOI:** 10.4084/MJHID.2013.008

**Published:** 2013-01-02

**Authors:** Giovanni D’Arena, Roberto Guariglia, Oreste Villani, Maria Carmen Martorelli, Giuseppe Pietrantuono, Giovanna Mansueto, Giuseppe Patitucci, Emilio Imbriani, Tommaso Masciandaro, Ludovica Borgia, Giulia Vita, Fiorella D’Auria, Teodora Statuto, Pellegrino Musto

**Affiliations:** 1 Department of Onco-Hematology, IRCCS “Centro di Riferimento Oncologico della Basilicata”, Rionero in Vulture, Italy; 2 Pathology Unit, IRCCS “Centro di Riferimento Oncologico della Basilicata”, Rionero in Vulture, Italy; 3 Urology Unit, IRCCS “Centro di Riferimento Oncologico della Basilicata”, Rionero in Vulture, Italy; 4 Laboratory of Clinical Research and Advanced Diagnostics, IRCCS “Centro di Riferimento Oncologico della Basilicata”, Rionero in Vulture, Italy

## Abstract

We report a patient with chronic lymphocytic leukemia (CLL) in whom a leukemic involvement of prostate and penis occurred in the advanced phase of his disease. Obstructive urinary symptoms were indicative of prostatic CLL infiltration, followed by the occurrence of an ulcerative lesion on the glans. Histologic examination confirmed the neoplastic B-cell infiltration. Both localizations responded to conventional treatments. A review of the literature confirms that leukemic involvement of the genito-urinary system is uncommon in CLL patients. However, it should be considered in CLL patients with urologic symptoms and a long history of the disease.

## Introduction

Chronic lymphocytic leukemia (CLL), the most common form of leukemia in adults in Western countries, is a B-cell neoplastic disorder characterized by a progressive accumulation of functionally incompetent CD19+, CD20+, CD5+, CD23+ and CD10− clonal lymphocytes. In the vast majority of patients, the disease mainly involves bone marrow, peripheral blood, lymph nodes and spleen. Extramedullary and extranodal involvement is rarely seen at diagnosis in these patients. However, especially in the end-stage disease, neoplastic cells can spread to non-hematopoietic sites and several reports have described a number of different anatomic sites that may be infiltrated by CLL cells.

Prostatic localization has been previously reported in CLL patients (Table 1)^1^–^7^ while penis involvement has been described, to the best of our knowledge, only in three patients so far (Table 2).^8^–^10^

Here we describe the unique case of a patient suffering from CLL who developed during his clinical history infiltration in both the prostate and the penis.

## Case Report

A 50-year-old man was diagnosed with an asymptomatic, immunologically typical (CD19+CD5+CD23+k+^low density^) CLL (Rai II clinical stage) in May 1999.

The patient underwent clinical and laboratory follow-up for more than 4 years. On August 2003, due to development of thrombocytopenia and an increase of lymphocytosis and spleen size, he was scheduled to receive 6 cycles of fludarabine plus cyclophosphamide (FC regimen) until to April 2004. A satisfactory response was obtained and maintained until July 2006, when the patient experienced again progressive lymphocytosis and splenomegaly, along with the appearance of diffuse nodal involvement and severe hypogammaglobulinemia. From August 2006 to January 2007, the patient received 6 cycles of rituximab, cyclophosphamide, prednisone, vincristine and liposomal doxorubicin (R-COMP regimen). A partial response was achieved. However, at the end of this treatment, three consecutive episodes of pneumonia occurred and the patient received multiple cycles of broad spectrum antibiotics, as well as monthly administrations of intravenous immunoglobulins.

**Figure 1:**
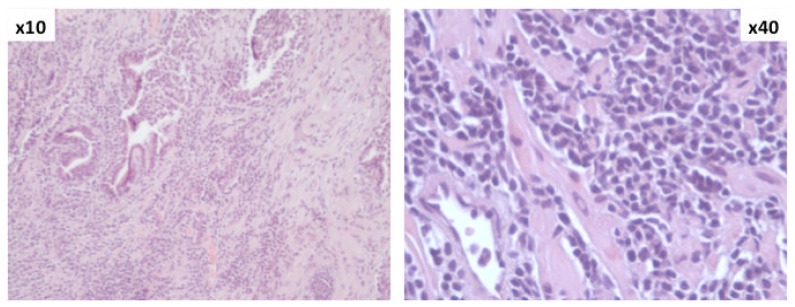
Histologic examination of prostate (HE stain, magnification ×10 and ×40): diffuse lymphocytic leukemic infiltration.

**Figure 2:**
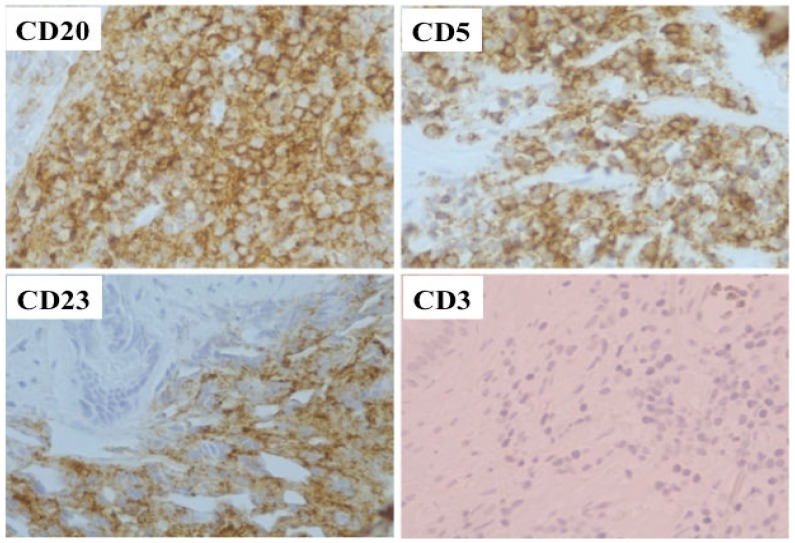
Histologic examination of prostate. Immunohistochemical stain (magnification ×40) showing diffuse positive staining of lymphocytes for CD20, CD5, CD23, and negativity for CD3.

On April, 2008, the patient complained of obstructive urinary symptoms; a “clinical” diagnosis of prostatic hyperplasia was made and the patient underwent a transurethral resection of the prostate. The histologic examination of the resected prostatic tissue showed a massive infiltration by small mature lymphocytes, resulting in a diagnosis prostatic involvement by CLL (Figure 1 and Figure 2). At that time, circulating lymphocytes were 34,000/μL and the spleen was 8 cm below the left costal margin. Prostatic irradiation (30 Gy) was performed and the obstructive urinary symptoms resolved. Due to further systemic progressive disease, the patient received chlorambucil for 6 months, followed by bendamustine for 6 additional cycles. A life threatening reaction to rituximab did not allow continuing this drug after a first infusion.

**Figure 3:**
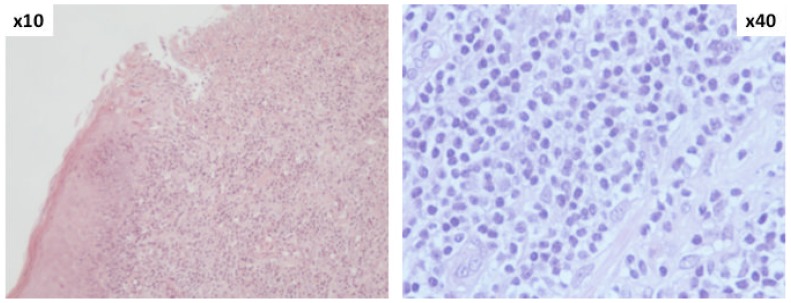
Histologic examination of subcutaneous tissue of penis. (HE stain, magnification ×10 and × 40): diffuse leukemic lymphocytic infiltration of subepithelial connective tissue with epithelial ulceration of penile mucosa.

**Figure 4:**
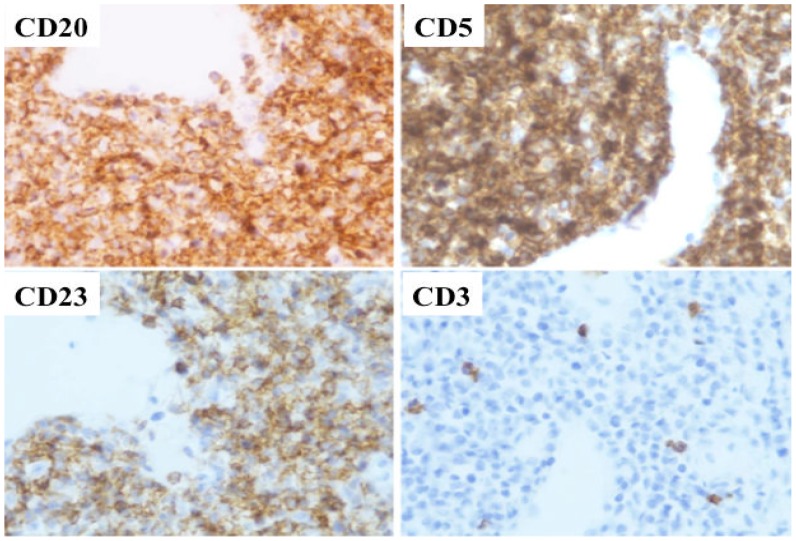
Histologic examination of subcutaneous tissue of penis. Immunohistochemical stain (magnification ×40) showing diffuse positive staining of lymphocytes for CD20, CD5, CD23, and negativity for CD3.

On November, 2010, the patient experienced an ulcerative lesion on the gland and a biopsy was performed. The histological workup showed again a lymphocytic infiltration (Figure 3 and Figure 4) and FC therapy was restarted. The penile lesion improved, but the patient died of acute respiratory distress on March, 2011, before starting the scheduled local penile treatment by radiotherapy.

## Discussion

Leukemic involvement of the genito-urinary system is uncommon in CLL patients. However, several reports of atypical leukemic infiltration by mature neoplastic B-cells have been published, the first in 1953.^11^ In 1973 Butler and O’Flynn reported that 6 out 4,863 patients who had undergone prostatectomy (0,12%) had prostatic involvement by leukemic cells and concomitant CLL.^1^ In addition, in a cohort of 5,962 subjects with several types of cancer in whom an autopsy was performed, Zein et al found that, among 88 cases with CLL, 18 (20,4%) showed persistent prostatic infiltration by leukemic cells.^2^

In another series, Terris et al reported about 1,092 patients who underwent radical prostatectomy and lymph node dissection; 13 patients (1,2%) were found to have low grade lymphoid malignancies,^4^ 3 of whom had CLL. In the majority of cases, such an infiltration was asymptomatic and had no impact on the life of patients. In other occasions, however, the infiltration by CLL causes urinary symptoms. This was also seen in our patient, where urinary outflow obstruction led to transurethral resection and the discovery of CLL infiltration.^12^,^13^

Radiation therapy is recommended as treatment of choice in these cases and it was effective in our patient too.

Secondary involvement of the penis, either by solid or hematologic neoplasms, is a very rare event. In particular, only 3 publications have so far reported such a localization in CLL patients.^8^–^10^ Systemic therapy induced local partial remission in our patient demonstrating sensitiveness of the lesion even after multiple treatments. Also for the penile localization the possibility of local radiotherapy should be taken into account.

To the best of our knowledge, this is the first report of CLL with evidence of both prostate and penis leukemic localizations. Due to rarity of these events, it is not possible to establish whether they may have prognostic relevance. However, genito-urinary involvement should be considered in CLL patients, mainly in those with urologic symptoms and a long history of the disease.

## Figures and Tables

**Table 1:** Published reports with more than 2 patients with CLL/SLL prostatic involvement and type of study.

Reference	Year	No. of cases	Type of study
Butler MR^1^	1973	6 CLL	Incidental diagnosis at the time of prostatectomy
Zein TA^2^	1985	18 CLL	Prostatic infiltration by leukemic cells in 18 (20,4%) out of 88 patients with CLL
Donohue^3^	1996	3 SLL	Concomitant diagnosis of SLL in patients with adenocarcinoma of the prostate
Terris MK^4^	1997	3 CLL 6 SLL	Lymphoid neoplasia diagnosed at the time of radical prostatectomy
Eisenberger CF^5^	1999	6 SLL	Incidental SLL in localized prostate cancer
Chu Pg^6^	2005	13 CLL/SLL	Incidental diagnosis at the time of prostatectomy
Schneiederjan SD^7^	2009	4 CLL/SLL	Retrospective clinicopathological study of 40 cases with lymphoid neoplasms of the urinary tract and male genital organs

**Table 2:** Published cases of CLL/SLL involving the penis and their clinical presentation.

Reference	Year	No. of cases	Clinical presentation
Gonzales-Campora R^8^	1991	1 SLL	Ulcerated thickening of shaft, painless priapism
Gatto-Weis C^9^	2000	1 CLL	Ulcerative balanoposthitis of the foreskin
Plaza JA^10^	2009	1 CLL	Erosive dermatitic eruption of the groin and penile shaft
